# LC-MS/MS analysis of plasma polyunsaturated fatty acids in type 2 diabetic patients after insulin analog initiation therapy

**DOI:** 10.1186/1476-511X-12-169

**Published:** 2013-11-06

**Authors:** Mutay Aslan, Filiz Özcan, Ibrahim Aslan, Gültekin Yücel

**Affiliations:** 1 Department of Medical Biochemistry, Akdeniz University Medical School, Antalya, Turkey; 2 Endocrinology Clinic, Antalya Research and Education Hospital, Antalya, Turkey

**Keywords:** Polyunsaturated fatty acids, Diabetes mellitus, Insulin, Prostaglandin

## Abstract

**Background:**

Eicosanoids derived from omega-6 (n6) polyunsaturated fatty acids (PUFAs) have proinflammatory functions whereas eicosanoids derived from omega-3 (n3) PUFAs have anti-inflammatory properties. This study was designed to evaluate the effect of insulin analog initiation therapy on n6 and n3 PUFAs in type 2 diabetic patients during early phase.

**Methods:**

Sixteen type 2 diabetic patients with glycosylated hemoglobin (HbA1c) levels above 10% despite ongoing combination therapy with sulphonylurea and metformin were selected. Former treatment regimen was continued for the first day followed by substitution of sulphonylurea therapy with different insulin analogs (0.4 U/kg/day) plus metformin. Blood samples were obtained from all patients at 24 and 72 hours. Plasma levels of arachidonic acid (AA, C20:4n6), dihomo-gamma-linolenic acid (DGLA, C20:3n6), eicosapentaenoic acid (EPA, C20:5n3) and docosahexaenoic acid (DHA, C22:6n3) were determined by an optimized multiple reaction monitoring (MRM) method using ultra fast-liquid chromatography (UFLC) coupled with tandem mass spectrometry (MS/MS). Prostaglandin E_2_ (PGE2) was measured in serum samples by enzyme immunoassay.

**Results:**

All measured PUFAs were significantly increased after treatment with insulin analogs plus metformin compared to before treatment levels. The mean AA/EPA ratio was significantly lower after treatment with insulin analogs plus metformin. A 22% decrease was observed in PGE2 levels after treatment with insulin analogs plus metformin compared to pretreatment levels (p > 0.05).

**Conclusion:**

The significant decrease in AA/EPA ratio indicates that insulin analog initiation therapy has anti-inflammatory properties by favoring the increase of n3 fatty acid EPA.

## Introduction

Increasing evidence supports the role of chronic inflammation in the pathogenesis of diabetes-associated microvascular and macrovascular complications [1]. Inflammatory cytokines, such as interleukin- 6 (IL-6), IL-1, and IL-18, as well as tumor necrosis factor- alpha (TNF-α), are involved in the development and progression of diabetic complications [2]. Preliminary results from clinical trials with salicylates and interleukin-1 antagonists support the hypothesis that the pathogenesis of type 2 diabetes mellitus (T2DM) reflects an inflammatory disorder [3]. Anti-inflammatory therapeutics simultaneously lower blood glucose levels, reduce the severity and prevalence of associated diabetic complications [4].

Polyunsaturated fatty acids (PUFAs) regulate inflammatory responses through the production of eicosanoids including prostaglandins (PGs), thromboxanes (TXs) and leukotrienes (LTs) [5]. The human body can produce many fatty acids except the two essential PUFAs which include linoleic acid (LA, C18:2n6) and alpha-linolenic acid (ALA, C18:3n3). Linoleic acid is the precursor of omega-6 (n6) series of PUFAs while ALA is the precursor of omega-3 (n3) series of PUFAs. Eicosanoids derived from n6 PUFAs such as arachidonic acid (AA, C20:4n6) have proinflammatory and immunoactive functions, whereas eicosanoids derived from n3 PUFAs such as eicosapentaenoic acid (EPA, C20:5n3) have anti-inflammatory properties, attributed to their ability to inhibit the formation of n6 PUFA-derived eicosanoids [5]. Resolvins and protectins generated from EPA and docosahexaenoic acid (DHA, C22:6n3) display potent anti-inflammatory properties and are recognized in the resolution of inflammation [6].

The beneficial effects of n3 PUFA, has been shown in obese-diabetic mice [7]. Omega-3 PUFA enriched diet increased expression of genes involved in glucose transport [glucose transporter type 4 (GLUT-4)] and insulin signaling [insulin receptor substrate 1 (IRS-1)], as well as genes involved in insulin sensitivity [peroxisome proliferator-activated receptor gamma (PPARγ)] [7]. There is also evidence showing that use of n3 PUFA supplementation improves clinical outcomes of patients with diabetes. In a diabetic and insulin-resistant state, n3 PUFAs bind to the G-protein coupled receptor 120 (GPR120), resulting in reduced cytokine production from inflammatory macrophages and improved signaling in adipocytes, leading to a reduction in insulin resistance [8].

Insulin stimulates the conversion of essential fatty acids (LA and ALA) to longer-chain PUFAs [9]. Indeed, levels of the principal n6 PUFA, AA, are reported to be significantly lower in diabetic patients than in controls [10,11]. Moreover, the contents of n3 PUFAs are decreased in serum and platelet lipids of diabetic patients compared to healthy controls [12]. It was hypothesized herein that insulin analog initiation therapy may increase long chain PUFAs and change AA/EPA ratio which is a marker of silent inflammation. Thus, this study was designed to evaluate the effect of insulin analog initiation therapy on n6 and n3 PUFAs in type 2 diabetic patients during early phase.

## Materials and methods

### Patients

The study group included 16 patients who were admitted to Antalya Research and Education Hospital, Endocrinology Clinic with a diagnosis of T2DM. Patient characteristics and laboratory values are shown in Table 1. The body mass index (BMI) of all patients enrolled in the study was <30 kg/m^2^ and all were non-smokers. None of the patients received antilipidemic agents in the last 3 months before the study. Subjects with apparent history of stroke, coronary heart disease, peripheral artery disease, severe kidney dysfunction, liver disease, thyroid dysfunction, infectious disease, and malignancy were excluded. All subjects enrolled were maintained on a standardized diet before the initiation of the study. HbA1c levels in all patients were above 10% despite ongoing therapy with sulphonylurea and metformin for at least 3 months. Former treatment regimen was continued for the first day followed by substitution of sulphonylurea therapy with insulin analogs. Patients received either 0.4 U/kg/day lispro mix (50% insulin lispro protamine and 50% insulin lispro) subcutaneously (SC) in three equal doses plus 2000 mg/day oral metformin; or 0.4 U/kg/day insulin glargine SC in one dose plus 2000 mg/day oral metformin. The given insulin treatments were in accordance with American Association of Clinical Endocrinologists (AACE) Diabetes Mellitus guidelines [13]. All patients gave written informed consent prior to entry. This study was approved by the Institutional Review Board of Antalya Research and Education Hospital and was performed in accordance with the Declaration of Helsinki.

**Table 1:** Patient characteristics and laboratory values

**Variable**	**Mean ± SD**	**n**
**Age (years)**	54.88 ± 11.76	16
**BMI (kg/m**^**2**^**)**	25.22 ± 2.15	16
**HbA1c (%)**	11.79 ± 2.41	16
**BUN (mg/dl)**	12.56 ± 3.05	16
**Serum Creatinine (mg/dl)**	0.81 ± 0.17	16
**Microalbumin (mg/24h)**	26.69 ± 18.57	16
**ALT (U/L)**	20.75 ± 10.60	16
**AST (U/L)**	24.00 ± 10.40	16
**TSH (μU/L)**	1.13 ± 0.64	16

BMI, body mass index; HbA1c, hemoglobin A1c; BUN, blood urea nitrogen; ALT, alanine aminotransferase; AST, aspartate aminotransferase; TSH, thyroid stimulating hormone.

### Laboratory measurements

Blood was obtained from all patients at 24 and 72 hours. HbA1c levels were determined by Abbott ARCHITECT c16000 System (Abbott Diagnostic, Abbott Park Illinois, USA) via immunoturbidimetric method. Total cholesterol (TC), HDL-C and triacylglycerol (TG) were measured on Roche Cobas 8000 Modular Analyser (Basel, Switzerland) via enzymatic colorimetric methods. Low-density lipoprotein cholesterol (LDL-C) and very low-density lipoprotein cholesterol (VLDL-C) levels were calculated via the Friedewald formula [14]. Blood urea nitrogen (BUN), serum creatinine, alanine aminotransferase (ALT), and aspartate aminotransferase (AST) were measured on Roche Cobas 8000 Modular Analyzer via colorimetric methods. Serum thyroid stimulating hormone (TSH) and urine microalbumin was measured on Roche Cobas 8000 Modular Analyzer via electrochemiluminescence immunoassay and turbidimetric methods, respectively.

### Electrospray ionization mass spectrometry

Standards for AA (C20:4n6), DGLA (C20:3n6), EPA (C20:5n3) and DHA (C22:6n3) were purchased from Sigma-Aldrich (St. Louis MO, USA). Deuterium labeled AA-d8 internal standard (5,6,8,9,11,12,14,15-AA-d8) was obtained from Santa Cruz Biotechnology (Santa Cruz, CA, USA). Solutions of AA, DGLA, EPA, DHA and AA-d8 standards were prepared in analytical grade methanol (Merck, Darmstadt, Germany). An optimized multiple reaction monitoring (MRM) method was developed using ultra-fast liquid chromatography (UFLC) coupled with tandem mass spectrometry (MS/MS). A UFLC system (LC-20 AD UFLC XR, Shimadzu Corporation, Japan) was coupled to a LCMS-8040 triple quadrupole mass spectrometer (Shimadzu Corporation, Japan). Chromatographic separations were carried out using Inertsil HPLC column (ODS-4, 2.1×100 mm, 3 μm; GL Sciences Inc. Tokyo, Japan) maintained at 40°C. DHA, EPA, AA and DGLA were separated using a gradient elution with a flow rate of 0.45 ml/min. Mobile phase solvent A was 10 mM ammonium acetate (Sigma-Aldrich, St. Louis, MO, USA) in water and solvent B was acetonitrile (Sigma-Aldrich, St. Louis, MO, USA). Gradient program was solvent B, 70% (0 min), 90% (3 min), 100% (3.01-4 min) and 70% (4.01-8 min). MRM transitions and responses were automatically optimized for individual compounds in negative electrospray ionization (ESI). In the negative ESI-MS mode the precursor and product m/z values for AA, DHA, EPA, DGLA and AA-d8 are given in Table 2. Responses to AA, DHA, EPA and DGLA were optimized to a linear calibration range from 100 ng/ml to 30 ug/ml and a sample analysis time of 8 minutes.

**Table 2:** The precursor and product m/z values for analyzed polyunsaturated fatty acids

	**Precursor m/z**	**Product m/z**
**DGLA (C20:3n6)**	304.80	59.00, 260.70
**AA (C20:4n6)**	303.10	59.00, 258.90
**EPA (C20:5n3)**	301.10	59.10, 256.70
**DHA (C22:6n3)**	327.10	59.10, 283.20
**AA-d8**	311.10	59.10, 97.90, 267.10

DGLA, dihomo-gamma-linolenic acid; AA, arachidonic acid; EPA, eicosapentaenoic acid; DHA, docosahexaenoic acid.

### Sample preparation for LC-MS/MS

Samples were prepared for LC-MS/MS analysis via a modified protocol as previously described [15]. Briefly, in a glass test tube, 200 μl plasma was added to 200 μl AA-d8 internal standard solution. 1 ml of acetonitril/37% hydrochloric acid (Cayman, Ann Arbor, MI, USA) was added to the mixture in a 4:1 v/v. Tubes were capped with reusable teflon liner screw caps and samples were hydrolyzed by incubating at 90°C for 2 hours in a heating block (VLM, Bielefeld, Germany). After cooling down to room temperature, fatty acids were extracted with 2 ml of hexane. Samples were vortex-mixed for 20 seconds, left at room temperature for 5 minutes and centrifuged at 3000 rpm for 1 minute. The upper phase containing free fatty acids were transferred to glass tubes and evaporated at room temperature under a constant stream of nitrogen with height adjustable gas distribution unit (VLM, Bielefeld, Germany). Fatty acids were dissolved in 200 μl methanol–water (180:20, v/v) filtered via 0,2 μm polytetrafluoroethylene (PTFE) syringe filters (Whatman, GE Healthcare Bio-Sciences, Pittsburgh, USA) and transferred to autosampler vials (Vertical Chromatography, Nonthaburi, Thailand).

### Measurement of prostaglandin E_2_

Prostaglandin E_2_ (PGE2) was measured in serum samples by a commercial enzyme immunoassay test kit [KGE004B; R&D Systems, Inc., Minneapolis, MN 55413, USA] according to manufacturer’s instructions. Briefly, PGE2 present in the sample competes with horseradish peroxidase (HRP)-labeled PGE2 for binding sites on a mouse monoclonal antibody. PGE2 in the sample is allowed to bind to the antibody in the first incubation. During the second incubation, HRP-labeled PGE2 binds to the remaining antibody sites. Following a wash to remove unbound materials, a substrate solution is added to the wells to determine the bound enzyme activity. The color development is stopped, and the absorbance is read at 450 nm. The intensity of the color is inversely proportional to the concentration of PGE2 in the sample. A standard curve of absorbance values of known PGE2 standards was plotted as a function of the logarithm of PGE2 standard concentrations (pg/ml) using the GraphPad Prism Software program for windows version 5,03. (GraphPad Software Inc). PGE2 concentrations in the samples were calculated from their corresponding absorbance values via the standard curve.

### Statistical analysis

Data were analyzed using Sigma Stat (version 2.03) statistical software for Windows, and a P value < 0.05 was considered statistically significant.

## Results

### Blood glucose and lipid profile

Mean blood glucose, TG and VLDL-C levels were significantly decreased while HDL-C levels were significantly increased after treatment with insulin analogs plus metformin compared to before treatment levels (Table 3). No significant decrease was observed in TC and LDL-C levels after treatment with insulin analogs plus metformin. Statistical analysis was done by paired t-test. The observed decrease in TG concentration and the increase in HDL-C support previous observations on the effect of insulin on plasma lipid levels [16].

**Table 3:** Mean blood glucose and lipid profile before and after insulin analog initiation therapy

**Parameter**	**Before treatment (mean ± SD)**	**After treatment (mean ± SD)**	**n**	**p value**
**Glucose (mg/dl)**	229.31 ± 49.78	183.00 ± 57.25*	16	<0.001
**Total cholesterol (mg/dl)**	219.50 ± 42.97	207.81 ± 39.56	16	0.056
**TG (mg/dl)**	225.73 ± 68.42	191.40 ± 71.08*	15	0.025
**VLDL-C (mg/dl)**	45.15 ± 13.68	38.28 ± 14.22*	15	0.025
**LDL-C (mg/dl)**	141.52 ± 39.99	134.25 ± 39.01	15	0.175
**HDL-C (mg/dl)**	32.25 ± 8.16	35.31 ± 9.29*	16	0.006

TG, triglyceride; VLDL-C, very low-density lipoprotein cholesterol; LDL-C, low-density lipoprotein cholesterol; HDL-C, high-density lipoprotein cholesterol.*, p < 0.05.

### ESI-MS spectra

**Figure 1:**
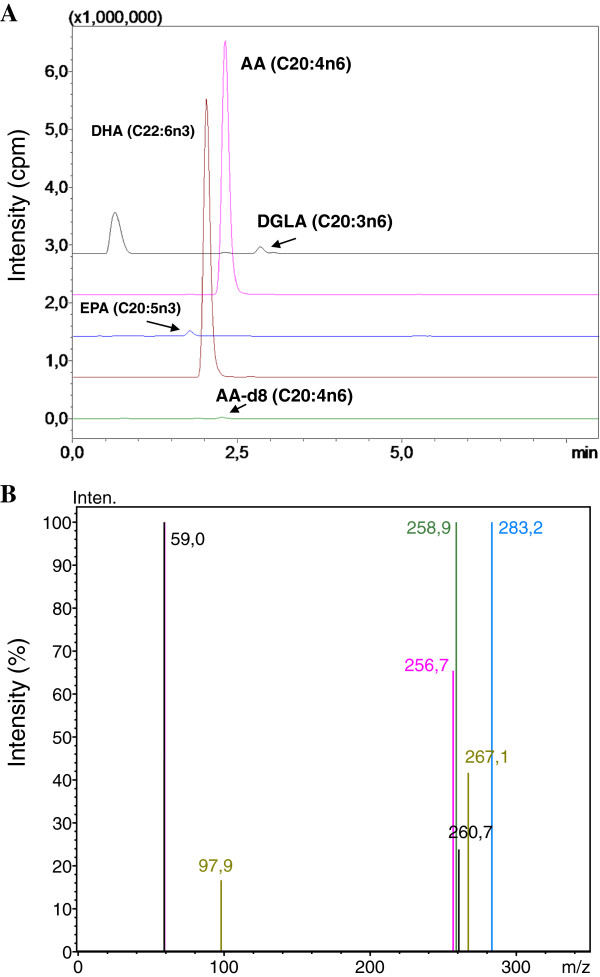
**ESI-MS Spectra. A)** Representative negative ion mode spectra of a patient sample. DGLA, Dihomo-gamma-linolenic acid; AA, Arachidonic acid; EPA, Eicosapentaenoic acid; DHA, Docosahexaenoic acid. **B)** Tandem mass spectra.

Figure 1A shows representative negative ion mode spectra of a patient sample. As shown in the figure, retention time of EPA (C20:5n3), DHA (C22:6n3), AA (C20:4n6), AA-d8 and DGLA (C20:3n6) was 1.869, 2.131, 2.391, 2.329 and 2.911 minutes. Figure 1B shows tandem mass spectra obtained by collision-induced dissociation of precursor ions. The m/z values of product ions correspond to endogenous C20:5n3, C20:4n6, C20:3n6 and C22:6n3. The deuterium-labeled internal standard fatty acid peaks are indicated at m/z values 97.9 and 267.1.

### Levels of polyunsaturated fatty acids

**Figure 2:**
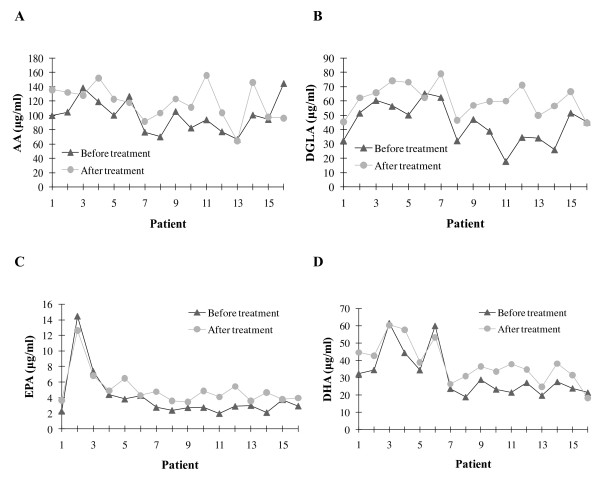
**Levels of polyunsaturated fatty acids in patients before and after insulin analog therapy. A)** AA **B)** DGLA **C)** EPA **D)** DHA. DGLA, Dihomo-gamma-linolenic acid; AA, Arachidonic acid; EPA, Eicosapentaenoic acid; DHA, Docosahexaenoic acid.

Levels of PUFAs before and after insulin analog therapy are given in Table 4. All measured PUFAs were significantly increased after treatment with insulin analogs plus metformin compared to before treatment levels. The mean AA/EPA ratio was significantly lower after treatment with insulin analogs plus metformin. No significant decrease was observed in AA/DHA ratio after treatment with insulin analogs plus metformin (Table 4). Statistical analysis was done by paired t-test. Comparison of AA (C20:4n6), DGLA (C20:3n6), EPA (C20:5n3) and DHA (C22:6n3) in serum samples before and after insulin analog therapy are shown in Figure 2.

**Table 4:** Analysis of polyunsaturated fatty acids before and after insulin analog initiation therapy

**Parameter**	**Before treatment (mean ± SD)**	**After treatment (mean ± SD)**	**n**	**p value**
**DGLA (C20:3n6) (μg/ml)**	44.11 ± 13.97	60.84 ± 10.72*	16	<0.001
**AA (C20:4n6) (μg/ml)**	100.10 ± 23.28	117.65 ± 24.66*	16	0.018
**EPA (C20:5n3) (μg/ml)**	3.98 ± 3.09	5.07 ± 2.25*	16	0.004
**DHA (C22:6n3) (μg/ml)**	31.33 ± 13.31	38.16 ± 11.66 *	16	<0.001
**AA/DHA**	3.47 ± 1.08	3.23 ± 0.77	16	0.065
**AA/EPA**	31.28 ± 11.76	25.34 ± 7.88*	16	0.006

DGLA, Dihomo-gamma-linolenic acid; AA, Arachidonic acid; EPA, Eicosapentaenoic acid; DHA, Docosahexaenoic acid.

### Prostaglandin E_2_ Levels

**Figure 3:**
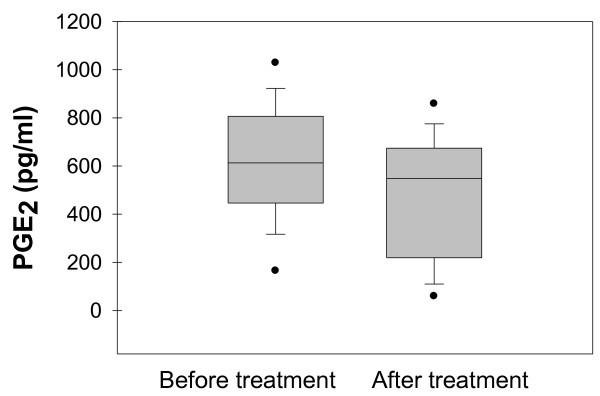
**Prostaglandin E**_**2 **_**Levels before and after insulin analog therapy.**

Box plot graph data of serum PGE2 content are shown in Figure 3. The boundary of the box closest to zero indicates the 25th percentile, the line within the box marks the median, and the boundary of the box farthest from zero indicates the 75th percentile. Whiskers above and below the box indicate the 90th and 10th percentiles.

Approximately 22% decrease was observed in PGE2 levels after treatment with insulin analogs plus metformin (p > 0.05). PGE2 (mean ± SEM) measured after treatment with insulin analogs (477.17 ± 66.58 pg/ml) was decreased compared to before treatment level (613.39 ± 63.79 pg/ml). Statistical analysis was done by paired t-test.

## Discussion

Altered availability of long chain PUFAs has been linked to the development of vascular complications and to the impairment of peripheral nerve function of diabetic patients [17]. We have seen that insulin analog initiation therapy plus metformin significantly increased plasma levels of AA (C20:4n6), DGLA (C20:3n6), EPA (C20:5n3) and DHA (C22:6n3) compared to before treatment levels. As far as we know, no report has addressed the effect of insulin analog initiation therapy on plasma PUFA levels in patients with T2DM.

Fatty acid composition of serum phospholipids were measured in 11 type 1 diabetic patients after a day of hyperglycemia and after normoglycemia was reestablished by insulin infusion. An increase was observed in AA, DGLA, EPA and DHA content of serum phospholipids after normoglycemia was reestablished as compared to hyperglycemic levels [18]. Similarly, plasma fatty acid composition was reported in 9 type 1 diabetic children during and after diabetic ketoacidosis (DKA) and an increase was observed in AA and DHA values after treatment of DKA [19].

Disturbed fatty acid metabolism is an important feature of the insulin-resistant state [20]. Essential fatty acids are metabolized into more physiologically active compounds by introduction of further double bonds by delta-5- and delta-6-desaturase enzymes [9]. The hepatic microsomal delta-6-desaturation of LA and ALA was found to be depressed in alloxan induced diabetic rats [21]. The observed enzymatic defect was corrected by insulin injection in 2 days [22]. It was demonstrated that blocking mRNA transcription by actinomycin D injection before insulin administration impaired the recovery of delta-6 desaturase activity suggesting that insulin induced the synthesis of delta-6 desaturase. Indeed it was later shown that delta-6- desaturase mRNA was 7 fold lower in streptozotocin-diabetic rat than in the control and the administration of insulin induced the enzyme mRNA 8 fold within 24 hours [23]. In vivo experiments also showed similar effects of diabetes on rat liver delta-5 desaturation and the correcting effect of insulin [24]. Animal studies investigating the role of type 2 diabetes on fatty acid desaturases are limited and not very clear. However reported data herein demonstrate for the first time that insulin analog initiation therapy in T2DM patients does increase long chain PUFAs in human plasma.

Another important finding of the presented work is that treatment with insulin analogs plus metformin significantly decreased the mean AA/EPA ratio compared to pretreatment levels. Competition between n6 and n3 fatty acids occurs in the production of eicosanoids by stereospecific lipid-oxidizing enzymes cylooxygenase (COX) and lipoxygenase (LOX) [25]. Eicosanoids, derived mainly from AA and EPA, are key mediators and regulators of inflammation. They include PGs, TXs, LTs and hydroxyeicosatetraenoic acids (HETEs) [26]. Eicosapentaenoic acid (C20:5n3) is a precursor of eicosanoids with less marked inflammatory effect. On the other hand, AA is a precursor of eicosanoids with definite inflammatory effect [27]. Hence, decreased AA to EPA ratio indicates less precursor for the synthesis of highly inflammatory eicosanoids.

Production of PGs occur by stereospecific lipid-oxidizing enzymes. Experimental animal models of diabetes have shown that PG synthesis is active in the presence of insulin deficiency [28] and that plasma and urine lipid oxidation are markedly decreased following insulin analog plus metformin treatment [29]. AA is a precursor of PGE2 synthesis and thus, decreased PGE2 levels following treatment with insulin analogs may also play a role in the observed increase of arachidonic acid.

Limitations of our study include (1) the study covers a small cohort 2) the study has not determined LA and ALA levels. Determining LA and ALA levels in patients enrolled in the study might have strengthened analysis of the data. Higher concentrations of LA result in a greater conversion of LA to arachidonic acid [30]. Similarly, increasing ALA intake results in significant enhancement in the EPA content of plasma phospholipids [31]. Decreasing LA in the diet to an optimal LA/ALA ratio of 4:1 results in higher plasma phospholipid EPA [32]. Around 8–20% of ALA is converted to EPA in humans, while conversion of ALA to DHA is less and predicted to be around 0.5–9% [33].

In conclusion, we have observed that treatment with insulin analog plus metformin resulted in a significant increase in long chain PUFAs and a significant decrease in AA/EPA ratio. These alterations may be of importance to understand the role of insulin analog therapy in decreasing the progression of chronic diabetic complications.

## Abbreviations

AA: Arachidonic acid; AACE: American Association of Clinical Endocrinologists; ALA: Alpha-linolenic acid; ALT: Aminotransferase; AST: Aspartate aminotransferase; BMI: Body mass index; BUN: Blood urea nitrogen; COX: Cylooxygenase; DGLA: Dihomo-gamma-linolenic acid; DHA: Docosahexaenoic acid; DKA: Diabetic ketoacidosis; EPA: Eicosapentaenoic acid; GLUT-4: Glucose transporter type 4; GPR120: G-protein coupled receptor 120; HbA1c: Glycosylated hemoglobin; HDL-C: High-density lipoprotein cholesterol; HETEs: Hydroxyeicosatetraenoic acids; IL: Interleukin; IRS-1: Insulin receptor substrate 1; LA: Linoleic acid; LDL-C: Low-density lipoprotein cholesterol; LOX: Lipoxygenase; LTs: Leukotrienes; MRM: Multiple reaction monitoring; MS/MS: Tandem mass spectrometry; n3: Omega-3; PGE2: Prostaglandin E_2_; PGs: Prostaglandins; PPARγ: Peroxisome proliferator-activated receptor gamma; PTFE: Polytetrafluoroethylene; PUFAs: Polyunsaturated fatty acids; SC: Subcutaneously; T2DM: Type 2 diabetes mellitus; TG: Triglyceride; TNF-α: Tumor necrosis factor- alpha; TSH: Thyroid stimulating hormone; TXs: Thromboxanes; UFLC: Ultra fast-liquid chromatography; VLDL-C: Very low-density lipoprotein cholesterol.

## Competing interest

All authors declare that they have no financial, consulting, and personal relationships with other people or organizations that could influence the presented work.

## Authors’ contributions

MA, carried out LC-MS/MS analysis, measurement of PGE2 and drafted the manuscript. FO, carried out LC-MS/MS analysis and measurement of PGE2. IA carried out the clinical studies including enrollment of patients, collection of blood samples and contributed in the drafting of the manuscript. GY assisted in mass spectrometry applications. All authors read and approved the final manuscript.
